# Cytomegalovirus, Macrophages and Breast Cancer

**DOI:** 10.2174/1874357901711010015

**Published:** 2017-03-31

**Authors:** S. Pasquereau, F. Al Moussawi, W. Karam, M. Diab Assaf, A. Kumar, G. Herbein

**Affiliations:** 1 Pathogens & Inflammation/EPILAB Laboratory, Department of Virology, University of Franche-Comté, COMUE Bourgogne Franche-Comté University, UPRES EA4266, SFR FED 4234, CHRU Besançon, Besançon, France; 2 Université Libanaise, Beyrouth, Lebanon

**Keywords:** HCMV, Tumor, Macrophages, Breast cancer

## Abstract

The human cytomegalovirus (HCMV) is a betaherpesvirus that is highly host specific, infects among others epithelial cells and macrophages, and has been recently mentioned as having oncomodulatory properties. HCMV is detected in the breast tumor tissue where macrophages, especially tumor associated macrophages, are associated with a poor prognosis. In this review, we will discuss the potential implication of HCMV in breast cancer with emphasis on the role played by macrophages.

## MACROPHAGES, HETEROGENEOUS CELL POPULATIONS

**Fig. (1):**
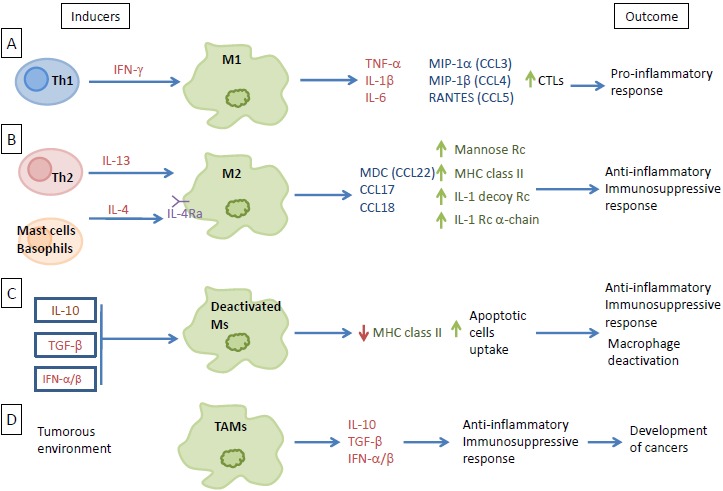
**Macrophages, heterogeneous cell populations.** (**A**) Th1 induction of M1 through interferon-gamma signaling. M1 releases specific cytokines and chemokines as part of the proinflammatory response. (**B**) M2 differentiation *via* IL-13 and IL-4 released from Th2, mast cells, and basophils. Differentiated M2 then releases factors that favor the anti-inflammatory and immunosuppressive responses. (**C**) Ms deactivation through the IL-10, TGF-beta, and IFN-alpha/beta signaling. Deactivation downregulates the expression of MHC class II and increases the uptake of apoptotic cells, resulting in an anti-inflammatory and immunosuppressive response. (**D**) Macrophages differentiation towards a specific phenotype present in the tumorous environment (TAMs), where they release large amount of immunosuppressive cytokines and a little amount of pro-inflammatory cytokines indirectly promoting the development of cancer. CTLs: Th1-cytotoxic T cells ; IFN: Interferon ; IL: Interleukine ; MHC: Major Histocompatibility Complex ; MIP: Macrophage Inflammatory Protein ; TNF: Tumor Necrosis Factor ; TAMs: Tumor Associated Macrophages ; Th: T helper ; Rc: Receptor ; CCL : C-C Motif Chemokine Ligand ; M : Macrophage ; MDC : Macrophage-Derived Chemokine ; RANTES : Regulated on Activation, Normal T cell Expressed and Secreted ; TGF : Transforming Growth Factor.

Against pathogens, macrophages (Ms) constitute the first defensive line of the organism where they become differentially activated in response to the microenvironment. Cellular immunity response to viral infection includes the well-established, classically activated macrophages (M1) that are induced by T helper 1 (Th1)-type responses through the interferon-gamma induction. This interferon (INF)-gamma induction of type 1 macrophages displays a proinflammatory profile with high levels of interleukin (IL)-1 beta, IL-6, tumor necrosis factor alpha (TNF alpha), and other proinflammatory cytokines (Fig. **1A**), in both of the plasma and lymph nodes, especially during systemic viral infections [1]. Classically, chemokines secretions are increased in patients with a M1 phenotype after viral infection. These chemokines include macrophage inflammatory protein (MIP)-1alpha, MIP-1beta and RANTES (CCL3, CCL4 and CCL5 respectively) (Fig. **1A**) [1, 2]. Immune activation is achieved by the increase of antiviral immunity with the enhancement of Th1 activity and the increase in IFN gamma, IL-12, IL-2 and IL-18 levels, especially in the lymph nodes of virally-infected subjects [3]. Therefore, M1 macrophages have a pivotal role in cellular immunity. They are involved in immunodeficiency syndromes, and in responses towards tissue damage and delayed-type hypersensitivity reactions [4]. Furthermore, M1 macrophages are important anti-cancer players. They stimulate the Th1-cytotoxic T cells (CTLs) and other effectors cells, thus improving patient survival. This makes them an important target that might be used to make a successful immunotherapy of cancers [5, 6].

Another activated macrophage (M2) differentiation pathway is possible in the presence of Th-2 type response and through the production of specific cytokines, such as IL-4 and IL-13 (Fig. **1B**). These M2-macrophages are part of the defense against parasitic infections, and display anti-inflammatory and tissue repair properties [4, 7]. Due to their participation in matrix-remodeling and their immunosuppressive activity, they can favor tumor growth. Macrophages may be activated alternatively by the action of IL-4 and IL-13 through a common receptor chain (IL-4Ra) (Fig. **1B**) [4, 8, 9]. IL-4, a pleiotropic cytokine, is produced by Th-2 cells, which constitute a specific subset of CD4+ T cells, and by basophils and mast cells [10]. IL-13, secreted by activated T cells, has been shown to induce major changes in human monocytes phenotype. Their antigen presenting capabilities are increased by upregulating the expression of multiple cell surface molecules. The effect of IL-4 and IL-13 on macrophages leads to the upregulation of the mannose receptor and MHC class II molecules expression which stimulate endocytosis and antigen presentation. This alternative activation pathway also upregulates macrophage-derived chemokine (CCL22) expression [8]. In addition, IL-4 and IL-13 counteract the pro-inflammatory actions of IL-1 through the increased expression of the IL-1 receptor alpha-chain and the IL-1 decoy receptor, *in vitro* and *in vivo* (Fig. **1B**) [4].

The deactivation of macrophages is mostly induced by the production of another cytokine: IL-10. IL-10 acts on a plasma membrane receptor that is distinct from those for IL-4 and IL-13 [7, 11]. Similar to IL-10, TGF-beta and IFNalpha/beta are also involved in macrophage deactivation with strong anti-inflammatory properties and downregulation of MHC class II molecules on the plasma membrane [7]. Macrophages deactivation leads to immune suppression, by creating an anti-inflammatory response, through the increase of apoptotic cells intake and the reduction of MHC class II molecules surface expression (Fig. **1C**) [12-14].

In the tumorous environment, macrophages will differentiate towards a specific phenotype, known as tumor-associated macrophages (TAMs) [15]. Most TAMs exhibit a phenotype close to M2 macrophages. After entering tumorous tissue near breast cancer cells, macrophages may acquire an M2 state. Immunosuppressive cytokines, *e.g.*, IL-10 and TGF-beta, are secreted in large amounts by these M2 macrophages, along with a little amount of pro-inflammatory cytokines (Fig. **1D**) (reviewed in [16]). These immunosuppressive M2 macrophages indirectly promote the development of cancers. In general, there is a correlation between the number of TAMs and poor prognosis, depending on the tumor type. In solid tumors, TAMs make up 5 to 40% of the tumor mass [15].

## MACROPHAGES, AN EXQUISITE TARGET FOR CYTOMEGALOVIRUS INFECTION

Human cytomegalovirus (HCMV) is a virus causing asymptomatic infection in healthy people and infecting a huge part of the population worldwide. It is an opportunistic, species-specific betaherpesvirus that can cause, in the absence of an effective immune response, severe disease, especially in patients with AIDS, in immunocompromised solid-organ and bone marrow allograft recipients, and in cancer patients. Immunohistochemical studies showed that HCMV infected cells can be present in virtually all body organs. A variety of cell types can be the *in vivo* host to the virus, including fibroblasts, macrophages, epithelial cells, stromal cells, smooth muscle cells, hepatocytes, endothelial cells and neuronal cells [17, 18].

A characteristic feature of the infection by HCMV is the persistence of the viral genome, as nonproductive form for months or even years, at specific anatomical sites in the normal host [19]. Several strategies enable the virus to avoid its elimination by the immune system, including: exploiting tissues that are immunologically privileged for replication (*i.e.*, epithelial cells of the salivary glands that express an insufficient amount of MHC Class I molecules to trigger the clearance by CD8+ cell), inducing a latent state of infection, and expressing genes interfering with the immune response [20]. HCMV also has the ability to exploit mechanisms interfering with chemokine-driven inflammation which enables the evasion from the immune response [21]. Four immunomodulatory glycoproteins, namely gpUS2, gpUS3, gpUS6 and gpUS11 are encoded by the human CMV. They decrease the expression of the major histocompatibility complex (MHC) class I proteins on the cell surface [22]. Proteins encoded by these four functional genes are dispensable for the replication of the virus but should impede the immune surveillance of cytotoxic CD8^+^ T lymphocyte (CTL). This occurs by reducing the MHC class I proteins levels on the infected cells surfaces [22, 23]. Furthermore, gpUS2 impedes the translocation of MHC class II proteins and could give CMV the ability to evade CD4^+^ T cell effector functions [24]. NK cells are also shown to be involved in the control of CMV infection [25]. In humans, normal cells are protected from NK cells by the expression of MHC Class I HLA–E molecules which bind to NK cells CD94 inhibitory receptors. HCMV UL 40 gene encodes for a protein, homologous to MHC Class I molecules, resulting in the up-regulation of Class I HLA-E molecules expression. This allows HCMV target cells to escape from NK cell attack [26].

The role of blood monocytes and tissue macrophages is central in case of infection. These cells serve as targets in the infected organs where they function either as viral disseminators throughout the host or as sites of HCMV latency [27]. The most prevalent infiltrating cell type found in HCMV-infected organs is the macrophage [28, 29]. Experimentally, fibroblast is the most widely used cell type to grow HCMV. It produces high titers of infectious virus after *in vitro* infection. Macrophages are also permissive for HCMV replication but in a lower manner than fibroblasts and the rate of viral production in these cells is considerably less [29, 30]. Macrophages infection either with clinical isolates (HCMV-DB) or with laboratory strains (AD169) had resulted in low-level sustained growth in comparison to fibroblasts. This indicates that HCMV can infect macrophages cultures, producing much lower viral titers in the culture supernatants as compared to fibroblasts [30]. In fact, macrophages produce only low levels of HCMV and murine CMV (MCMV) [19, 25, 26].

The difference in viral growth kinetics between macrophages (low growth) and fibroblasts (high growth) is neither due to differential attachment and adsorption of the virus [31] nor a difference in viral entry [30], although the early phases of viral cycle might be a contributing factor in HCMV cell tropism. The restriction of HCMV replication in some cell types is believed to be mainly dependent on a post entry block to viral gene expression [32]. Recently, it has been reported that several blocks occur in the viral cycle following HCMV infection of cancer cells [33]. The virus ability to inhibit macrophage differentiation is required for optimal viral replication, and might be a factor of the low rate of viral growth in macrophage cultures [34, 35]. In addition, studies showed that the presence of an intact ULb’ sequence in the genome of clinical HCMV isolates had lead to their efficient growth in macrophages, epithelial cells and endothelial cells [36-39]. On the other hand, this ULb’ region is lost in the HCMV laboratory strains which leads to the inefficient replication in macrophages and epithelial cells [37].This reflects the importance of testing clinical strains rather than laboratory ones to study HCMV replication and pathogenesis. Finally, although HCMV infection susceptibility is higher in M2 macrophages, HCMV also infects classically activated macrophages. HCMV establishes a productive and persistent infection in both types of macrophages, which then acquire similar features of classical activation and secrete high levels of pro-inflammatory cytokines and chemokines [40-42]. Moreover, during acute HCMV infection, the IE1 CMV protein is involved in the production of the TNF alpha cytokine [43].

**Fig. (2):**
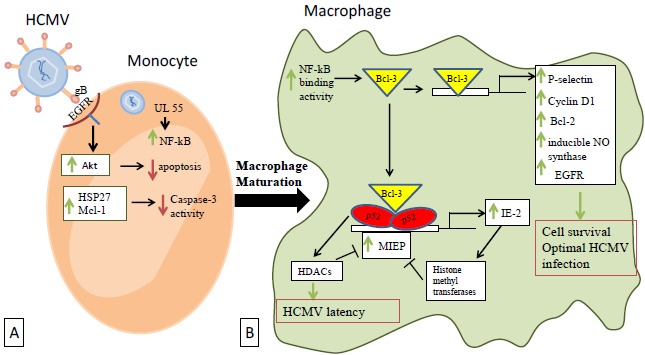
**Cellular transduction events occuring after macrophages infection by HCMV.** (**A**) HCMV binds to the cellular EGFR through its glycoprotein gB and directly activates Akt, thus promoting an Akt-dependent prosurvival state. EGFR activation rapidly induces the expression of HSP27 and Mcl-1 that act together to downregulate caspase-3 activity and permit the virus persistence. (**B**) In the differentiated macrophages, NF-kB binding activity is increased allowing Bcl-3 to activate a number of human genes that favor cellular survival and optimal infection by HCMV. Activated p52/Bcl-3 complexes also act through the NF-kB sites to regulate the MIEP of HCMV and thus affecting the virus latency *via* HDACs. MIEP is also under by negative autoregulation through IE2 expression which in turn activates histone methyltransferases. EGFR: Epidermal Growth Factor Receptor ; HDACs: Histone Deacetylases ; HCMV: Human Cytomegalovirus ; MIEP: Major Immediate Early Promoter ; Bcl: B-cell lymphoma encoded protein ; gB: Glycoprotein B ; HSP: Heat Shock Protein ; IE: Immediate Early ; Mcl-1: Induced myeloid leukemia cell differentiation protein ; NF-kB: Nuclear Factor-Kappa B.

Significant cellular signal transduction events occur in the HCMV-infected macrophages. During entry, HCMV glycoprotein gB binds to the cellular epidermal growth factor receptor (EGFR) initiating directly the activation of the apoptosis suppressor Akt (Fig. **2**). This activation promotes an Akt-dependent prosurvival state after monocytes infection [44]. In addition, HCMV also targets other signals in order to allow infected monocytes to pass the 48-hours cell fate decision checkpoint, which is required to begin maturation into macrophages. EGFR activation rapidly induces HSP27 and Mcl-1 expression (Fig. **2**), which function together to control caspase-3 activity precisely. Caspase-3 activity is a key process in the viral dissemination and it is responsible for permitting virus persistence [45]. The infection of monocytes differentiating into macrophages activates caspase-dependent or independent cell death programs. Both activated death programs are controlled by the HCMV UL36 gene [46]. In addition, the antiapoptotic Bcl-XL protein provides protection against CMV-induced apoptosis [47]. Furthermore, it is observed that in response to HCMV infection, NF-kB binding activity is increased in nuclear extracts of macrophages and fibroblasts [30]. Previous studies also reported that NF-kB is activated in response to HCMV infection in fibroblasts [48] and monocytes [49] through the HCMV UL55 (gB) ligands [49].

In addition to the NF-kB activation, p52/Bcl-3 complexes and the major immediate early promoter (MIEP) of HCMV are also shown to be activated after macrophage infection [30]. Through the NF-kB sites, activated p52/Bcl-3 complexes regulate the MIEP of HCMV, while Bcl-3 activates a number of human genes as reported by studies including P-selectin [50], cyclin D1 [51-54], Bcl-2 [55-57], inducible NO synthase [58], and EGFR [59, 60] (Fig. **2**). Most of the activated cellular genes favor cell survival to allow optimal infection by HCMV.

Histone deacetylases (HDAC) may play a role in HCMV latency in macrophages, although their possible recruitment by p52 [53] and Bcl-3 [61-63] is not fully characterized yet. In addition to HDACs, inhibition of MIEP activity by histone methyltransferases has been reported and could explain its negative autoregulation by IE2 (Fig. **2**) [64, 65].

In case of concomitant bacterial infection, HCMV enhances bacterial induction of macrophage inflammatory responses that are mediated through NF-kB pathway and thus promoting organ inflammation in HCMV-infected tissues [66, 67]. The comparison between HCMV-infected and mock-infected macrophages had lead to the observation that HCMV infection maintains CD14, TLR4 and TLR5 surface expression, which declines over time in mock-infected macrophages. IκBα and NF-κB phosphorylation is also enhanced, along with the expression of MyD88, an adaptor protein [67].

HCMV replication is poor in monocytes while it is enhanced in monocytes derived macrophages [45, 68]. When HCMV binds to monocytes, it induces an intracellular increase in Ca^2+^ levels. One of the results of the Ca^2+^ rise is the block of the monocytes ability to differentiate into macrophages. Observations suggest that the virus has an efficient strategy permitting it to interfere with cellular differentiation pathways, and this may also elucidate the generalized immunosuppression that is often observed in HCMV-infected patients [34, 69]. HCMV infection of CD14+ monocytes gives rise to the generation of latency-specific transcripts, conservation of viral genomes, and the capacity of the virus to reenter the lytic cycle. In addition, latent virus has an effect on the level of STAT1 phosphorylation leading to disruption in type I and II signaling [70].

## MACROPHAGES, CYTOMEGALOVIRUS AND CELLULAR TRANSFORMATION IN BREAST CANCER

Two distinct phenomena could be observed after HCMV infection of macrophages. First, the viral gene expression is regulated by the NF-kB switch, resulting in viral persistence through sustained low levels of viral replication. Additionally, HCMV-infected macrophages could fuel the progression of the disease by permitting the infection to spread to cells in the vicinity of infected macrophages, which could be more permissive cells, like fibroblasts and/or epithelial cells. During viremia, the virus present in blood could infect circulating monocytes [71, 72]. Upon migration of infected monocytes into breast tissue, they could, after their differentiation into macrophages, transmit the virus to the surrounding mammary epithelial cells [73]. Most of the breast cancers are carcinomas that have their origin at cells lining the milk-forming ducts of the mammary gland, especially transformed epithelial mammary cells. Moreover, these mammary epithelial cells lining the duct could be infected directly by HCMV present in the milk [74]. The infection of mammary epithelial cells by HCMV primary clinical isolates could favor their transformation (GH and AK unpublished data). Second, HCMV infection of macrophage could alter the expression of cellular genes in HCMV-infected cells resulting in an adjusted cellular phenotype, *e.g*., an M2 phenotype that will favor a protumoral microenvironment [30, 75].

Monocytes and macrophages constitute important HCMV reservoirs and their responsibility in the viral dissemination is well known [68, 72, 73]. Furthermore, HCMV infection of monocytes has the potential to reprogram them, giving rise to their polarization toward the inflammatory macrophages (M1), that also displays properties of immunosuppressive macrophages (M2) [75]. HCMV-infected monocyte transcriptome exhibits an unique M1/M2 polarization signature skewed towards the classical M1 activation phenotype [76]. This is mediated by induction of NF-κB and PI3K activities in these monocytes upon HCMV infection [30, 75]. M1 macrophages exhibit inflammatory cytokines expression, where their prolonged secretion is often connected with the development of cancer (reviewed in [77]). El-Shinawi and co-workers study has shown that the prevalence of HCMV IgG in patients with inflammatory breast cancer (IBC) is higher than that in non-IBC invasive ductal carcinoma (IDC) patients . They also observed that HCMV DNA levels are higher and NF-kB is more activated in cancerous tissues isolated from IBC in comparison with IDC patients. NF-κB enhanced activation can either be a result of HCMV infection of breast cells or of cytokine production in the tumor microenvironment [78, 79]. In another instance, we have observed in a patient oriented study that there is a positive correlation among the seroprevalence of HCMV IgG, elevated levels of IL-6, and the incidence of liver cancer [80].

Several attempts have been made to find a link between HCMV and the development of breast cancer. The large protein repertoire of the HCMV has the potential ability either to initiate or to promote neoplastic changes in cells. Richardson hypothesized that late exposure to HCMV could increase the incidence of breast cancer [81]. The basis for this hypothesis is the correlation between breast cancer incidence and HCMV seroprevalence. The correlation between levels of HCMV IgG and breast cancer development was investigated in a study conducted by Cox and colleagues, where they enrolled 399 invasive breast cancer patients and 399 control patients. Results showed a statistically significant correlation between HCMV IgG levels elevation and the development of breast cancer in women [82]. Furthermore, searching for HCMV in milk samples from HCMV seropositive women revealed that HCMV is present in more than 90% of the samples [83, 84], while the presence of HCMV DNA was also reported in normal breast tissue [85]. More direct evidence that the breast epithelium form an important reservoir for HCMV in humans was provided by the detection of HCMV antigens in breast biopsies. HCMV antigens prevalence was higher in the neoplastic epithelium of breast cancer patients as compared to normal breast tissue of breast cancer patients and non-cancer patients [86]. The presence of HCMV DNA and proteins both in breast cancer tissue and in sentinel lymph node metastasis tissue has also been recently reported [86, 87]. A model in which the HCMV infection of both mammary epithelial cells and macrophages initiates the transformation of epithelial cells in a favorable protumoral microenvironment could explain both the detection of HCMV DNA and/or antigens in breast tumors parallel to the presence of TAMs associated with poor prognosis.

**Fig. (3):**
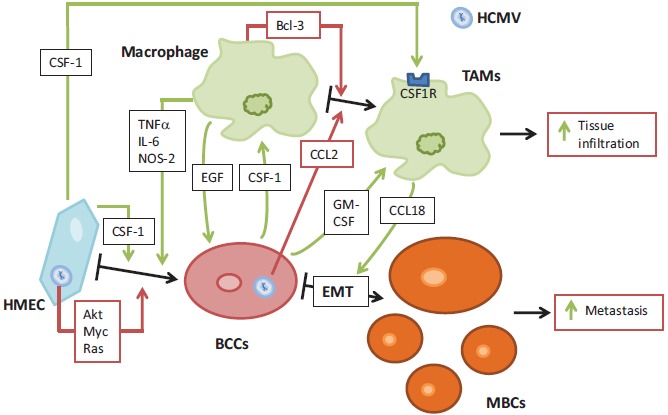
**Potential interplay between macrophages, mammary epithelial cells and HCMV in breast cancer.** HCMV infection of HMEC induces their priming towards epithelial transformation, by activation of several signaling pathways (Akt, Myc, Ras). HCMV also favors macrophages differentiation towards M2 phenotype. HMEC produces CFS-1, that will enhance cell transformation. This creates a favorable microenvironment, in which infected HMEC produce CCL-2 that promotes TAMs differentiation. In turn, TAMs secrete EGF, that promote breast cancer cells proliferation. Once HMEC had been transformed, they produce GM-CSF, which activate macrophages. In turn, TAMs secrete CCL18, that promotes the epithelial-mesenchymal transition (EMT) of breast cancer cells. This interplay favors cells activations and transformations, leading toward increased metastasis of breast cancer cells and increased tissue infiltration by TAMs. BCCs: Brest Cancer Cells ; CSF: Colony Stimulating Factor ; EMT: epithelial-mesenchymal transition ; HMEC: Human Mammary Epithelial Cells ; MBCs: Metastatic Breast Cancer cells ; TAMs: Tumor Associated Macrophages ; Bcl: B-cell lymphoma encoded protein ; CCL : C-C Motif Chemokine Ligand ; EGF: Epidermal Growth Factor ; GM: Granulocyte Macrophage ; HCMV : Human Cytomegalovirus ; IL: Interleukin ; NOS: NO Synthase ; TNF: Tumor Necrosis Factor.

*In vitro,* HCMV promotes oncogenic transformation in human mammary epithelial cells (HMECs) with activation of several signaling pathways such as PI3K/AKT, Myc and Ras (AK and GH, unpublished data). In addition, HCMV favors the activation of macrophages toward a M2 phenotype with increased Bcl3 activity (Fig. **3**) [30]. The priming of HMECs toward epithelial transformation and of macrophages toward M2 phenotype could create a favorable tumor microenvironment for breast tumor formation. Furthermore, HMECs secrete CSF-1 in high levels that could promote the proliferation of breast cancer cells [88]. The replication of HCMV can induce CCL2 secretions in fibroblasts and myeloid cells [89-91]. In turn TAMs can secrete EGF that bind to EGFR on the breast cancer cells. The development of a transformed phenotype in human breast cancer cells is enhanced by the benign mammary epithelial cells [92]. Finally, the invasion of breast cancer cells is stimulated, in culture, in the presence of human mammary fibroblasts, which increase, in mouse xenograft experiments, the development of stroma [93]. This will further promote the transformation of breast cancer cells and ultimately could lead to the development of breast adenocarcinoma.

Once HMECs had been definitively transformed, there will be no more requirements for the presence of HCMV that initiated the transformation, so it will be cleared from the breast cancer cells. In fact, we and others have reported that HCMV cannot replicate efficiently in transformed cells such as hepatocellular carcinoma HepG2 cells and in fibroblasts expressing SV40 T antigen and oncogenic H-Ras [18, 33]. HCMV replicates in a low level in M2 (TAM) macrophages because of the binding of a Bcl3-p52 NF-kB complex on the MIEP promoter that result in a reduced viral transcription (Fig. **2**) [30]. Altogether, this model of mammary epithelial cell transformation could rely on a « hit and run » theory in which HCMV (alone or with other prooncogenic factors) could promote the appearance of breast adenocarcinoma through a dual control of HMEC transformation and M2/TAM shift that will feed the initiation of the tumor which will then no more depend on HCMV existence and take its future in its own hands. Targeting TAMs has been proposed as a new therapeutic approach to fight breast cancer [94, 95].

It has been demonstrated that macrophages, as part of the tumor stroma, promote breast cancer cell migration and stem cell activity [96]. The co-culture of breast cancer cell lines (BCCLs) with THP-1 cell lines showed that migration of ER-positive breast cancer cell lines increased with all types of macrophages. And in this model of monocyte-macrophage differentiation, an increase in mammosphere formation occurs with M2-macrophages in comparison with M1-macrophages. In fact, HCMV favors the stemness of transformed cells with formation of tumorospheres in cellular models of glioblastoma, hepatocarcinoma and colon cancer *in vitro* [18, 96-100]. These results indicate that HCMV could favor the appearance of a more aggressive phenotype with increased risk of metastasis and poorer prognosis. Basal-like breast cancer (BBC) does not have any biologically targeted therapy yet, while it is considered an aggressive subtype of breast cancer. Important determinants of the tumor biology are the interactions of the stromal cells with the BBC cells. Inflammatory cells associated with the stroma play a key role in the progression of cancer. BBC cells co-culture with monocytes-like THP-1 cells have resulted in gene expression alteration with upregulation of both M1 and M2 markers. In relative to luminal breast cancers, the differentiation of monocytes to macrophages increased in BBCs with enhanced macrophage migration. It is observed that a distinct pattern of cytokines is secreted in macrophage-BBC co-cultures, including upregulation of NAP-2, MIG, MCP-1, MCP-3, osteoprotegerin, and interleukin (IL)-1β [101].

The epithelial-mesenchymal transition (EMT) of cancer cells that are present at the invasive front of tumors, in a close vicinity with TAMs, suggest that a mutual interaction might be present between these two cell types [15, 102]. Macrophages are activated to TAM-like phenotype by the effect of mesenchymal–like breast cancer cells *via* GM-CSF. CCL18 from TAMs induces cancer cell EMT and increases cancer metastasis. In breast cancer samples, the expression of GM-CSF in high levels is associated with the increase in CCL18(+) macrophages and the realization of EMT by cancer cells, which both translate into an increase of metastasis and a reduction of patient survival. This suggests the importance of a positive feedback loop between GM-CSF and CCL18 in breast cancer metastasis (Fig. **3**) [103].

The overexpression of macrophage colony-stimulating factor (CSF-1) and its receptor (CSF1-R) has been correlated to poor prognosis in human breast carcinomas. CSF-1R expression is restricted, at the tumor site, to macrophages, allowing the regulation of infiltration and function of TAMS by CSF-1. This action of CSF-1 could enhance the metastatic potential of tumor [104]. For their comigration and invasion into collagen I, which involves a paracrine loop, it is necessary and sufficient for macrophages and tumor cells to be present. Epidermal growth factor (EGF) is first expressed by macrophages and increases the formation of elongated protrusions and cell invasion by carcinoma cells. CSF-1 produced by carcinoma cells enhances the expression of EGF by macrophages. Moreover, EGF promotes CSF-1 expression by carcinoma cells thereby generating a positive feedback loop (Fig. **3**) [105]. Infiltration and activation of macrophages have been studied by immunohistochemistry in human primary breast tumors, from a large cohort. A poor differentiation and a fast proliferation was associated with high numbers of M2-macrophages in tumors showing estrogen receptor negativity and histological ductal type [106].

Finally, although HCMV infection of macrophages might favor a protumoral microenvironment, it could also result in the containment of the viral dissemination. In a murine model, peripheral MCMV infection spreads *via* lymph nodes and MCMV infects filtering macrophages, which support virus replication poorly. When these macrophages are depleted, MCMV infects susceptible fibroblasts and spread faster [107]. Also, depletion of splenic macrophages significantly enhances, rather than deters, replication of MCMV in the spleen [107]. Thus, tissue macrophages may protect other highly permissive cell types present in their vicinity from CMV infection parallel to the enhancement of a stemless protumoral environment, which could be critical in breast cancer and dissemination. Finally, once the CMV has played its role as an initiator and/or enhancer of tumorogenesis, viral expression within transformed cells not only will be limited ([33], GH and AK unpublished data), but also could limit cellular transformation. In fact, recent reports indicate that HCMV and MCMV infection could favor the clearance of tumoral cells [108, 109].

## CONCLUSION

A primary feature of breast cancer is a deleterious inflammation. Accumulating evidence demonstrates that macrophages have a critical role at each stage of cancer progression, as they are, in mammary tumors, the most abundant leukocyte population. These tumor-associated macrophages (TAMs) facilitate neoplastic transformation, are responsible for the tumor escape from immune responses and could lead to the subsequent metastatic cascade. HCMV infects macrophages and favors the appearance of a M2 phenotype, close to the TAM phenotype, parallel to the activation of oncogenic pathways in mammary epithelial cells. Breast tumors could induce and exploit, with the help of HCMV, trophic macrophages in order to subvert immune responses, avoiding the destruction of malignant cells. Therefore, macrophage- and/or HCMV-targeted intervention strategies have to be evaluated in order to curtain breast cancer morbidity and mortality.
